# Promotion of Wild Food Plant Use Diversity in the Soviet Union, 1922–1991

**DOI:** 10.3390/plants11202670

**Published:** 2022-10-11

**Authors:** Gayana Bexultanova, Julia Prakofjewa, Matteo Sartori, Raivo Kalle, Andrea Pieroni, Renata Sõukand

**Affiliations:** 1 Department of Environmental Sciences, Informatics and Statistics, Ca’ Foscari University of Venice, Via Torino 155, 30172 Venezia, Italy; 2 Department of History, University of Concepción, Edmundo Larenas 240, Concepción 4030000, Chile; 3 University of Gastronomic Sciences, Piazza Vittorio Emanuele II 9, 12042 Pollenzo, Italy; 4 Medical Analysis Department, Tishk International University, 100 Meter Street and Mosul Road, Erbil 44001, Iraq

**Keywords:** propaganda, wild food plants, history of botany, promotion, ethnobotany, Soviet Union, plant use knowledge, book knowledge

## Abstract

In the Soviet Union, wild food played a secondary role in diet (as cultivated species dominated). Yet the authorities eventually acknowledged their importance as diet diversifiers and a safety reservoir, and started to promote their use through various means, including publishing books on the use of wild food plants. These government publications appeared during a specific time, and therefore, we mapped all centralized publications in order to understand the dynamics of the promotion of wild-plant-related knowledge. For deeper analysis, we selected a sample of 12 books promoting wild food plants, and compared the taxa and uses represented in these works, which fall into two key periods: during World War II (1941–1943) and after the war (1953–1989). A total of 323 plant taxa belonging to 69 plant families were named, of which Rosaceae had the highest number of proposed food uses, prompting the reader to explore the use of borderland species. Most diverse food uses were attributed to *Sorbus aucuparia*, followed by *Rosa* and *Vaccinium oxycoccos*. Wartime books had fewer taxa with less variety, with a clear preference for staple food and substitutes, while post-war books promoted desserts and alcoholic drinks.

## 1. Introduction

Wild food plants (generally considered species that grow spontaneously in self-sustaining populations outside cultivated areas and used for nutrition) have been and still are a part of local diets all over the world [1,2,3]. While in the modern world, especially in industrialized parts, wild food plants have shifted to the periphery of food systems, though they still constitute a reservoir of healthy food, especially when food supplies are disrupted [4]. Therefore, they have recently become a widespread research topic [5,6,7].

Knowledge of the use of plants has historically been acquired through trial and error and subsequently circulated through oral and written communication between generations, keeping use associated with local flora. Wild plants constitute a significant food resource, especially in places highly vulnerable to climate change or military conflicts, although displacements of people erode local ecological knowledge which negatively contribute to food security [7,8,9]. Wild food plants were occasionally promoted in Europe during World War II (WWII) (see, for example, Finland [10], Norway [11]), yet after this time of hardship, the propagation of wild food plants in post-war Europe has been uncommon. An exception to this is Finland, where, starting in 1942, plant scientist Toivo Rautavaara (1905–1987) published 13 editions of his book on wild food plants. The last edition [12] also reached the post-Soviet space, and was translated into Estonian in the first years of regaining the independence [13]. In the Soviet Bloc, wild food plant guides were reserved for army survival camp handbooks (see, for example, Czechoslovakia [14]). Thus, promotions of the use of wild food plants in previous centuries often failed, despite the famine of the 19th century (see, for example, [15,16]), as they did during WWII in Central Europe (e.g., [17]). Some successful examples of wild food plant introduction through using these books do exist (e.g., [18]).

The USSR, as a country, encompassed several climatic and geographical zones with numerous ecological regions. The flora of the Soviet Union consisted of more than 17,000 higher plant species belonging to more than 160 families [19]. World War II (1941–1945) and the Soviet strategy significantly impacted food security, resulting in the reduced availability of agricultural products and food. The Soviet Union was formed de jure as a communist federal union, linking various republics together [20]. There was apparent centralized governance, with Moscow as the capital and control center [21]. 

Early Soviet policy promoted the development of individual non-Russian linguistic and cultural groups [22]. Starting in 1938, the Soviet government began to adhere to the policy of universalizing the spread of the Russian language. The policy shifted to more active propaganda on the use of the Russian language as a lingua franca, followed by brutal colonial Russification, particularly in the Soviet education system [23]. Russian was the publication language of the most widespread and influential scientific books. Soviet ideology [24] and Soviet propaganda [25] had a strong influence on the population, and, even in post-Soviet countries, there are still echoes of the influence of Soviet policies on the circulation of wild food plant knowledge in books [26].

In June 1941, war began in the territory of the Soviet Union after an attack by Nazi Germany. That same year, the 872-day siege of Leningrad (present-day Saint Petersburg) began, which caused disruption to all supplies, including food. Inevitably, this led to official reductions in the rations of ordinary citizens [27,28]. During the siege, a widespread campaign to promote wild edible plants was launched in the region [29]. In 1943, upon the decision of the Bureau of the Leningrad Regional Committee of the All-Union Communist Party of Bolsheviks, a plan for the procurement of wild food plants was approved [29]. In addition, a series of brochures and books on wild food plants were published and disseminated [29]. The materials for these books were prepared by researchers at the Komarov Botanical Institute (KBI) of the Russian Academy of Sciences [30]. It was one of the major academic institutions contributing to the botanical sciences during the Soviet era. At the beginning of the war, the work of the KBI was aimed at supporting the needs of national defense and, later, the population of besieged Leningrad [30]. The researchers actively studied the practical use of plants for food and medicinal purposes, analyzing their chemical compositions and characteristics obtained from the literature and laboratory experiments. As a result, the list of plants rich in vitamins was expanded, with a particular interest in vitamin C content [30].

After the war, most KBI staff previously involved in military actions returned to work. Initially, they mainly published delayed books and materials. 

Verzilin’s 1946 book “*Пo следам Рoбинзoна*” (“In Robinson’s footsteps”) was the earliest of the post-war books studied [31]. At that time, Verzilin worked in Leningrad [32], where he was head of the botany and zoology teaching methodology laboratory. The book became very popular and famous among the general public in the USSR, and was reprinted several times in the following years [33,34,35], remaining the only publication on the subject until the late 1960s. 

Regardless of their legacy, no attempts have thus far been made to understand the extent to which the use of wild food plants was popularized [36,37,38] on a central level through printed sources in the former USSR.

The precise temporal and geographical scope of our novel study allowed for extensive analysis to understand the ways in which wild food plants were presented as resources to the population. Therefore, the aim of the study was to provide an overview of popular books about the use of wild plants for food, centrally published in the Soviet Union. To this end, the following specific objectives were set:

(i) to select a representative sample of books that promoted the use of wild food plants in the Soviet Union;
(ii) to identify the most widely promoted taxa in them, and discuss their methods of preparation and food uses;
(iii) to describe the most relevant changes in knowledge circulation potentially occurring within the 70-year period (1921–1991).

## 2. Results

There were no centralized publications on wild food plants until 1941. For the purposes of analysis, we identified seven books and brochures published during the war period (between 1941 and 1945) and five books published after the war (1946–1989) (Table 1). The selected books were representative for the periods, had high print volumes, and were accessible full-text publications. 

A total of 2895 detailed use records (DUR) were identified, reflecting the use of 323 plant taxa belonging to 69 plant families. Of the 323 plant taxa, 77 (23.8%) were promoted by three or more different authors. After generalization, the analysis yielded 42 proposed food use categories from the 1098 food uses originally proposed by the authors.

### 2.1. Dominant Taxa in the Selected Books

**Figure 1:**
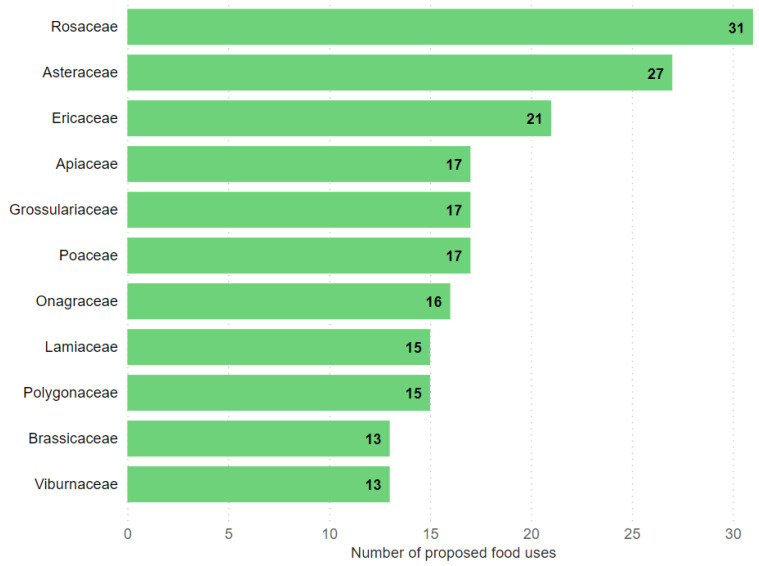
The top 11 plant families according to the number of proposed food use categories in all 12 books published centrally in Russian during the Soviet era.

Across all 69 reported families, the number of proposed food uses ranged from one to 31 per plant family. The top eleven plant families by the number of proposed food uses are shown in Figure 1.

As illustrated above, members of the family Rosaceae had the highest number of generalized proposed food uses among all plant families in the sources studied, with 31 uses. Next, with 27 proposed food uses, there was the Asteraceae family, followed by Ericaceae (21).

**Figure 2:**
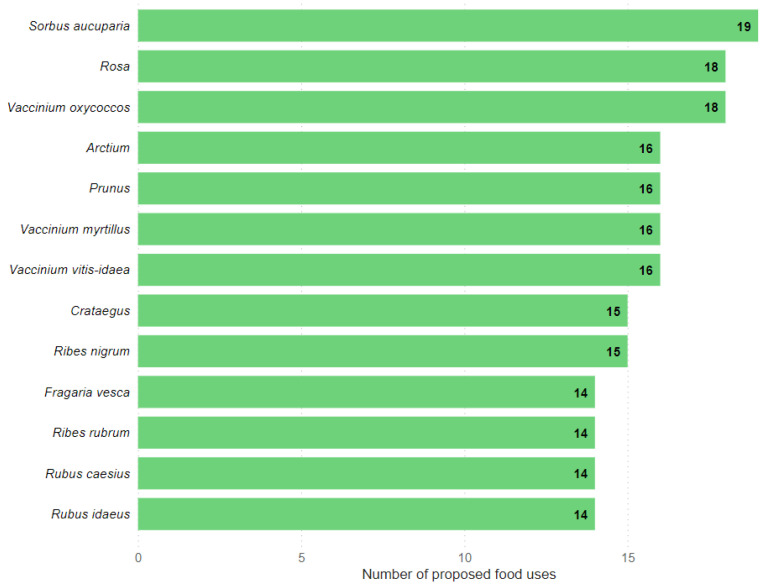
The top 13 plant taxa according to the number of proposed modes of food use in the aforementioned books.

Then, the number of proposed food uses of plants was analyzed. Figure 2 illustrates the plant taxa with 14 or more documented uses.

Out of the 77 taxa that were reported by three or more different authors, *Sorbus aucuparia* had the highest number of proposed food uses, with 19 uses, followed by *Rosa* and *Vaccinium oxycoccos*, with 18 proposed uses each. Next, *Arctium*, *Prunus*, *Vaccinium myrtillus*, and *Vaccinium vitis-idaea* were third, with 16 uses each.

**Figure 3:**
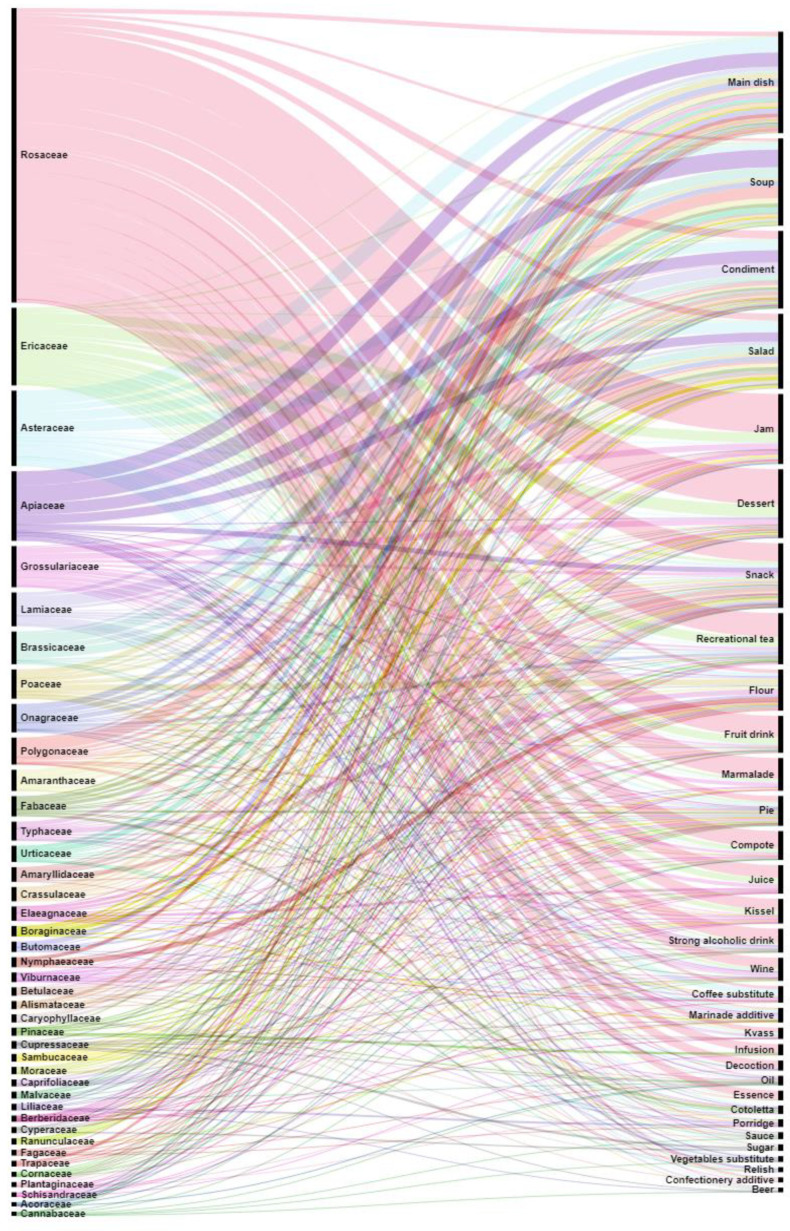
Alluvial diagram of the DURs for plant families with proposed food uses from the studied books.

The plant families were also analyzed, using RAWGraphs, on the basis of DURs to track the diversity of use and the comparative number of records (Figure 3).

### 2.2. Main Methods of Preparation

**Figure 4:**
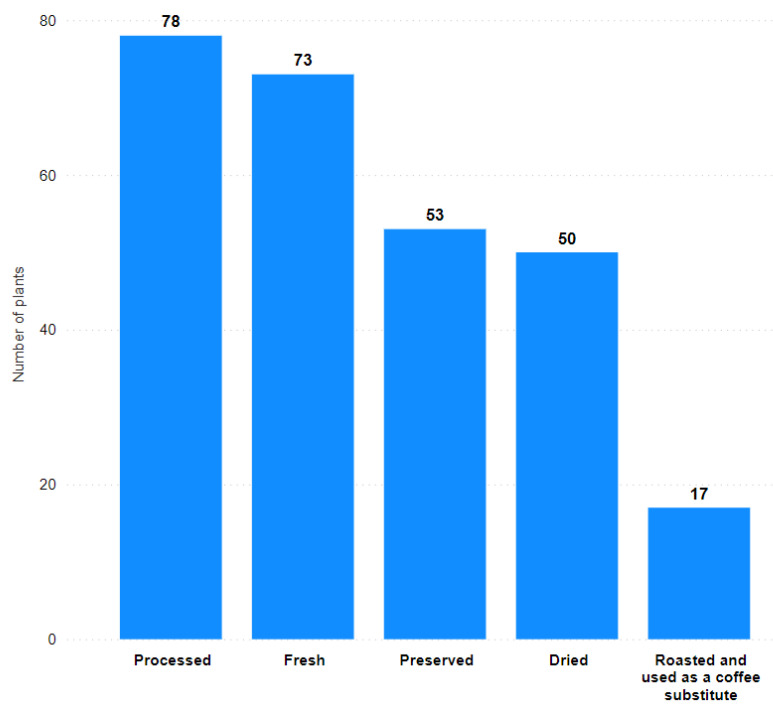
The ratio of preparation methods by the number of plant taxa.

Processed as a preparation method accounted for 28.8% of plant species named by at least three authors (Figure 4). In addition, 26.9% of the plant taxa were recommended to be consumed fresh.

**Figure 5:**
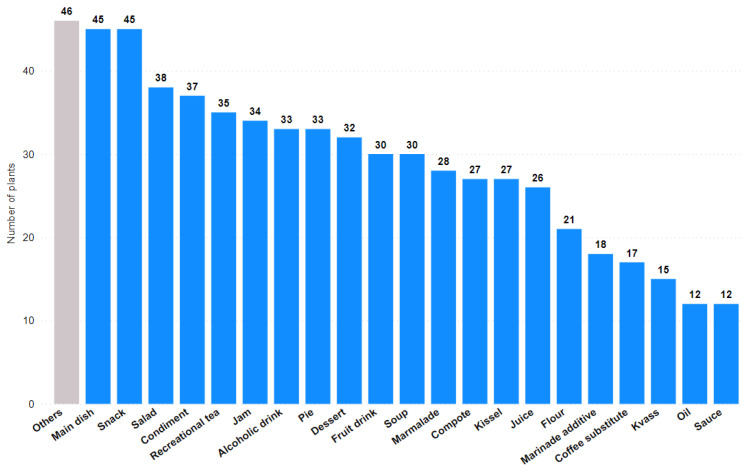
The most frequently proposed modes of food use in terms of the number of plant taxa.

The top three food uses identified in the literature were: as a main dish, as a snack, and in a salad (45, 45, and 38 plant taxa, respectively) (Figure 5). A fairly large number of plants were used as condiments (37), for recreational tea (35), and for making jam (34). The least common uses analyzed were the extraction of oil and the preparation of sauce (12 each).

### 2.3. Differences in the Selected Books between the Key Periods: During WWII (1941–1943) and after the War (1953–1989)

**Figure 6:**
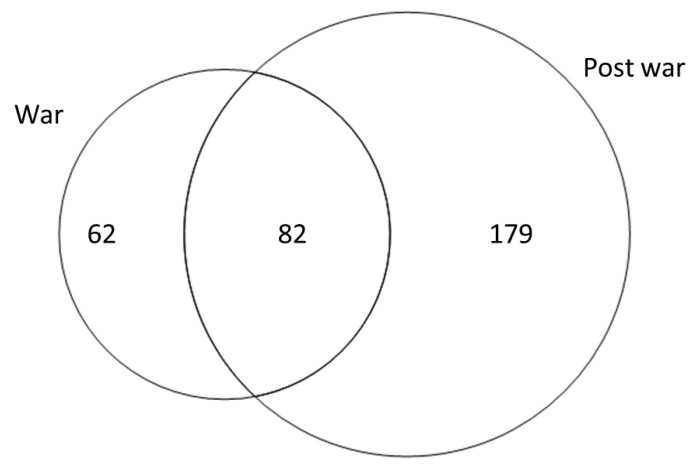
Venn diagram comparing the number of taxa promoted during the war and in post-war period, and the overlapping taxa.

A total of 144 wild food plant taxa circulated between 1941 and 1943. In comparison, between 1953 and 1989, 261 plant taxa were promoted in the books, which is 1.8 times higher than the number of plant species proposed for use during WWII (Figure 6). The war period was characterized by the promotion of leafy vegetables (*Aegopodium podagraria*, *Urtica*, *Chenopodium*, *Rumex acetosa*), while the post-war period promoted actively fruiting taxa (*Fragaria*, *Ribes*, *Rubus*, *Vaccinium*, *Rosa*). Of all promoted taxa, only 82 were shared between the two periods. Notably, among the most promoted taxa in either period, only three taxa overlapped (*Epilobium angustifolium*, *Rumex acetosa*, and *Typha latifolia*) (Table 2).

While the majority of the presented taxa are native, the few introduced taxa are represented unevenly in the two periods. During the war, only seven non-native taxa where introduced, while the post-war period contained 35 introduced taxa, many of them native to southern parts of the Soviet Union. Two non-native taxa in the northern part of the Soviet Union overlapped for both periods (*Morus* and *Borago officinalis*). 

Among all the taxa proposed for use, there were a number in either period (19 taxa for the war period and 48 for the post-war period) for which the exact use mode was not specified. For the war period, such non-specified taxa were often presented with indications of their vitamin and mineral content, while in the post-war period mainly various subspecies and less-common taxa were mentioned, including 13 non-native taxa. Interestingly, *Sorbus aucuparia*, which was proposed with the most diverse use in the post-war period, was only mentioned in passing in a war-time book [49] as a source of vitamin C.

**Figure 7:**
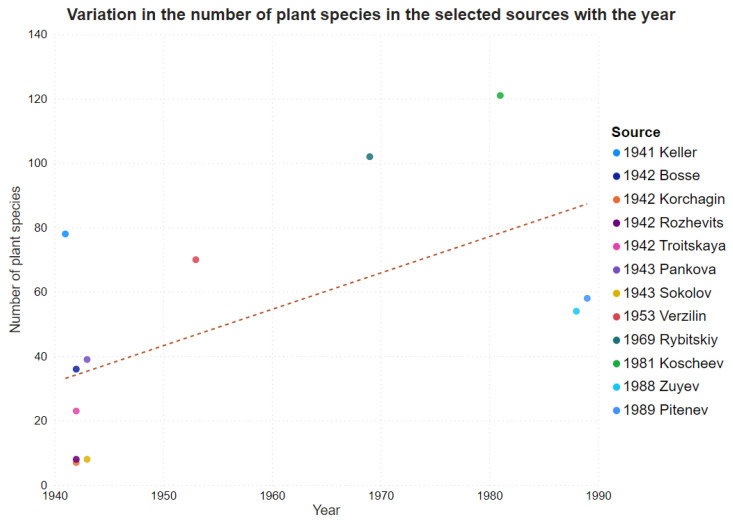
The relationship between year and the number of plant species in the sources.

The number of taxa discussed in the books was higher during the post-war period (Figure 7). 

A total of 41 generalized use modes of plants were reported during the post-war period, while only 28 were mentioned during WWII. Additionally, four selected food uses were analyzed to compare the results for the wartime and post-war periods (Table 3).

The DURs related to the main dish category were higher for the period during WWII, while in the post-war period desserts, substitutes, and alcoholic drinks dominated (Table 3).

## 3. Discussion

### 3.1. Dominant Taxa in the Selected Books

Rosaceae led the ranking by a significant margin, followed by Asteraceae and Ericaceae. These results are consistent with the results of field research: Rosaceae and Asteraceae were found to be frequently used by respondents in Europe [3], including many southern regions once occupied by the USSR [6,59], while Ericaceae, followed by Rosaceae, were predominant in northern regions [60,61,62]. The dominance of Rosacea could be seen as an invitation to explore more borderland species. 

The list of the top thirteen plants with the most proposed food uses includes seven members of the family Rosaceae, including *Sorbus aucuparia*, *Rosa*, and *Prunus*, which are among the top five plant taxa. Notably, only one member of the family Asteraceae (genus *Arctium*) was found on the list of plants with the highest number of proposed food uses. This can be explained by the fact that the family Asteraceae has a large number of plant taxa (28) which, although not included in the top list, leads to a large number of different food uses as a whole (27, Figure 1). The abundant use of the family Ericaceae (top three families in terms of the number of proposed food uses) is supported by three representatives of the family in the top thirteen used plants: *Vaccinium oxycoccos*, *V. myrtillus*, and *V. vitis-idaea* (Figure 2). This result can be attributed to the fact that 3,900,000 km^2^ of former Soviet territory is covered by the East Siberian taiga (boreal forest), where the forementioned plant taxa are the dominant shrubs [63,64], and therefore constitute a considerable resource which was popularized.

Almost all of the top thirteen plants were fruiting plants (the exception being the genus *Arctium*). The fruits were suggested to be used as snacks and condiments and for making recreational tea, jam, pies, desserts, various fruit drinks, marmalade, alcoholic drinks, etc. The top three plants all have fruits or berries. The genus *Sorbus* (mainly represented by *Sorbus aucuparia*) is largely present in the Northern Hemisphere [65], as do *Vaccinium oxycoccos*, *V. myrtillus*, and *V. vitis-idaea*, which also grow in the Northern Hemisphere [66], and members of the genus *Prunus* found in northern temperate regions [67].

### 3.2. Main Methods of Preparation

Most wild plants were consumed in processed form (cooked or baked), accounting for 28.8% of all plants (Figure 4). Parts of wild plants are often processed before consumption to improve their taste or to make them edible [2]. In addition, according to several authors [31,40], correct processing eliminates the toxic properties of plants by reducing or breaking down unwanted chemical substances. 

A fresh method of consumption was also very frequently suggested for wild plants (26.9%), which mainly included use as a snack or salad. For some green parts and fruits (berries), the recommended use in raw form can be explained by the fact that it is the easiest way to obtain the necessary nutrients and vitamins. Some studies, however, have shown that the stability of vitamins, and thus their accessibility for an organism before and after processing, depends on both the plant species and the geographical location [68]. 

Preserved, as a preparation method, was intended to conserve the plant for storage and future use, avoiding its spoiling. Typical methods of preserving wild food plants in the books of the Soviet Union included fermentation, lacto-fermentation, salting, drying, and storing with sugar. Preserved plants were used as condiments and desserts, and to prepare main dishes and soups, various salads, drinks, and recreational tea.

The three most commonly suggested food uses were main dishes, snacks, and salads (Figure 5). This may also be related to the problematic supply of basic foods in the USSR during both the war and the post-war period [69]. A voucher system was introduced in the USSR several times due to shortages of food and other goods. Vouchers set a certain rate of consumption of goods per person per month and allowed a one-time purchase of some goods [70]. There is a possible link between the voucher system in the USSR and the high number of plant species used to make recreational tea, as tea was one of the goods distributed under the voucher scheme. Thus, promotion of the use of wild plants for food by the Soviet government was aimed at reducing food shortages and increasing food security in the region.

### 3.3. Changes in Plant Use Knowledge in Soviet Books from the Key Periods: During WWII and after the War until the Fall of the USSR

On average, the number of plant species proposed for food use in the USSR during World War II was almost half the number of plant species recommended for use during the post-war period. This difference is related to the specificity of wartime books, some of which were solely dedicated to plant species from the Leningrad region or those potentially available in besieged town settings. Similarly, the number of proposed food uses of wild plants was found to be more than 46% higher for the post-war period compared to the wartime period. Moreover, during WWII, wild plants were not often used as desserts due to the more acute problem of basic food shortages, which also explains the dominance of the main dish category of staple foods, which was necessitated by wartime demands. Similarly, alcoholic drinks like wine, beer, and vodka were not a top priority during the war: 4 DURs for the wartime period compared to 134 DURs in the post-war period. Numerous plant parts were mentioned by the authors as substitutes for vegetables or spices during both periods. During the war, substitutes were reported for 22 DURs, which is significantly higher compared to the dessert and alcoholic drink categories. This was also due to the food supply crisis and the need to promote possible food substitutes that could be found in the forest or marshes close to home.

On average, the number of plants described for food use in the studied sources increased as the years progressed. One possible explanation for this trend is that over time, with the development of botany and science in general, scientific knowledge about the chemical composition and practical uses of certain plants for food increased [71]. Additionally, we should also consider other factors. For instance, changes in the availability of knowledge as well as human and natural resources which were developed over time and in particular places. One of the factors responsible for changes in book knowledge circulation might be the decreasing social and economic conditions until the fall of the USSR. However, the high number of taxa popularized during the 1970s and 1980s can perhaps be explained by the wide promotion of the wild food plant “richness” of the Soviet Union, which also introduced non-native taxa to the region of publication. 

From the analysis of book knowledge circulation in the USSR (1941–1989), we can observe the fluctuating interest in promoting wild food plants by Soviet scholars. The high number of books published during the siege of Leningrad was clearly motivated by specific needs, while in the period from 1946 to 1968, only the updated prints of one single book were published, and so this latter period we can consider quite a barren one. The last 22 years of the Soviet Union saw the publication of six original books. We can note an increase in taxonomic species and uses of wild plants during the studied period (except for the two last books), as well as the growth of diversity. 

It is important to add that the books published in Leningrad during the blockade were most likely not distributed widely throughout the country, and thus did not have much influence on the books published towards the final years of the Soviet Union.

## 4. Materials and Methods

### 4.1. The Definition of ‘Wild’ Plants Used in the Study and Plant Taxonomy

In the context of this study, wild food plants are plant species that were signaled as such by the authors of the books we studied.

The lack of accepted botanical nomenclature in the literature led to an additional analysis that entailed the use of the Plants of the World Online database (POWO) [72] and plant families following the Angiosperm Phylogeny Group. Each plant name reported by the author of a book was assigned an accepted binomial name. In cases where the exact plant species could not be identified, the genus of the plant was indicated. Often, the same plant name in Russian referred to different plants and sometimes to entire groups [73]. Therefore, we tried to identify as accurately as possible the plants described in the sources.

### 4.2. Geopolitical Area

**Figure 8:**
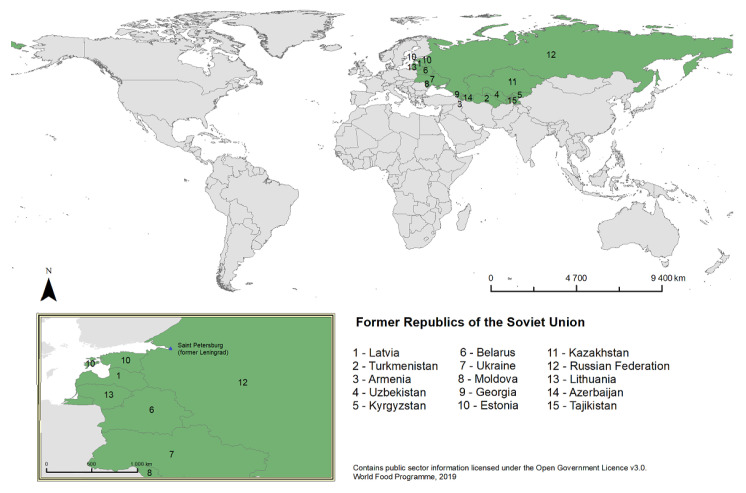
The studied region on the world map today.

The Soviet Union covered an area of 22,402,200 km^2^ [74]. Figure 8 shows the territory of the USSR along with the various republics that it encompassed.

The territory of the USSR stretched over different climatic zones [75] and various ecological regions, including tundra, taiga (boreal forest), temperate grasslands, savannas and shrublands, montane grasslands and shrublands, deserts, and xeric shrublands. The western part of the Soviet Union, including the city of Leningrad (present-day Saint Petersburg) and the Leningrad region, consists mainly of taiga and temperate broadleaf and mixed forests [76].

According to statistical data, the overall population of the Soviet Union was 195.4 million in 1941, dropping sharply to 170.5 million in 1946 as a result of WWII [77]. Based on Soviet censuses, the population of the USSR grew steadily in the following years until its collapse, reaching 208.8 million in 1959, 262.4 million in 1979, and 286.7 million in 1989 [78].

### 4.3. Data Sample

A preliminary search of the available literature on wild plants used for food purposes in the USSR was carried out using the National Library of Russia [79], the National Electronic Library of the Russian Federation [80], the Russian State Library [81], and the WorldCat library catalog [82].

A thorough review of the available books on wild plants used for food purposes in the USSR was conducted. Keywords used in the search included terms such as: “*дикие съедoбные растения СССР*” (“wild edible plants of the USSR”), “*дикoрастущие пищевые растения CCCP*” (“wild-growing food plants of the USSR”), “*cъедoбные дикoрастущие растения СССР*” (“edible food plants of the USSR”). The primary selection was based on the time period, region, and language of the source. Books in the national languages of the various Soviet republics, other than Russian (the official language of the USSR), were excluded. 

The sources selected were mostly scanned copies of books in electronic format. Some of the books were hard copies, which were then digitized for analysis by scanning. All the books retrieved were reviewed as possible sources of data. In addition, in order to facilitate the analysis of the most influential books in terms of potential readership, sources with a circulation of more than 3000 copies were highlighted. The next criterion concerned the content of the books. Books focusing on specific topics regarding the use of wild plants were excluded from the selection (e.g., “*Примитивные спoсoбы выпечки хлеба в пoлевых услoвиях*” (“Primitive ways of baking bread in the field”) [47]).

Of particular interest were books from the World War II period published between 1941 and 1945. Seven books from that period were included in this study, including Keller [39], Bosse [40], Korchagin [43], Rozhevits [45], Troitskaya et al. [46], Pankova and Nikitin [48], and Sokolov [49]. Other books [33,53,54,55,56] were selected for analysis on the basis of the year of publication.

### 4.4. Data Analysis

Microsoft Excel 2016 and Microsoft Power BI Desktop 2022 were used to analyze the obtained data.

The database containing information on the [83] was created as the product of a Master’s thesis by the first author [84]. The contents of the books were digitized and stored in a Microsoft Excel spreadsheet where each row represented a detailed use record (DUR), the structure and name of which were adopted from Sõukand and Kalle [85]. The DUR included the original information from the source: plant name in Russian, binomial name (if specified), plant family (if specified), availability of plant images, chemical composition (if specified), collection method, plant part used, mode of use, place of growth, if specified, region (e.g., Siberia) and location (e.g., swamp), collection time, and specific comments. 

For the purposes of this study, the information obtained was further processed by structuring and assigning new generalized categories. The original preparation methods were grouped into broader categories: processed, fresh, preserved, dried, or roasted and used as a coffee substitute.

The primary categorization of uses taken from the books was too broad to allow comparison between the written sources, and therefore the data were further generalized. For example, berries consumed fresh were described in the database as a snack (rather than as a *dessert* or another category). In addition, when the plant was used as a substitute for vegetables or spices in soups or main dishes, it was classified as a substitute because it was important to examine how books of the time presented information on food and vegetable substitutes due to the lack of a regular food supply.

The number of plant taxa was calculated, in Microsoft Excel, for each time period as well as for each plant family. More attention was given to taxa promoted by three or more authors. 

Next, an analysis of the proposed modes of food use was carried out using Microsoft Power BI. The number of proposed food uses was calculated for each plant family and plant taxon to identify those with the highest number of proposed food uses according to the authors.

For the purposes of the analysis, the category NS (not specified) was excluded from the estimate of the number of plant taxa prepared using a specific method. This category was found to be common to many plant taxa (i.e., for about 13% of the plant taxa the authors did not specify the exact preparation method or food use).

In order to more effectively assess the most frequently proposed food uses, categories, of which there were a large number, that contained 10 or fewer plant taxa were grouped into the others category. 

The plant species reported by the authors were analyzed according to the number of sources mentioning the plant. In those cases where the name of the plant was explicitly mentioned by the author (i.e., if the author reported both the genus and the species name or reported the scientific name of the plant in Russian), a binomial name in Latin could be assigned to it. In other cases, the plant was identified to the most precise possible level (often to genus).

When a plant with a generic name was reported to have a proposed food use by three or more authors, the genus was left unchanged. Similarly, when a plant with a binomial name was reported to have a proposed food use by three or more authors, the binomial name of the plant was used for the analysis. 

An analysis of the number of recorded plant species was carried out to compare the two periods of interest: during WWII (books published between 1941 and 1943) and after the war (sources from the period 1953–1989). The calculation included all plants mentioned in the books, including those that had no specific use indicated. In addition, due to the availability of data from four editions of Verzilin’s book [31,33,34,35], only the 1953 edition [33] was included in the calculation of the average number of plant species. This source was chosen because it is the median book between the three editions and refers to the ten-year period between 1950 and 1959. The change in the number of plants with a specific food use in the sources by year from 1941 to 1989 was determined by constructing a line of best fit. In addition, it was documented whether the studied books contained an image of the plant.

## 5. Conclusions

In 12 books selected from 24 books centrally published during the existence of the Soviet Union, 323 plant taxa belonging to 69 plant families were promoted. The Rosaceae plant family had the highest number of proposed food uses, prompting the reader to explore the use of borderland species. The most diverse food uses were promoted for *Sorbus aucuparia*, followed by *Rosa* and *Vaccinium oxycoccos*. Wartime books had fewer taxa with less variety, with a clear preference for staple foods and substitutes, while post-war books promoted desserts and alcoholic drinks.

In the circulation of book knowledge, wild plants were considered an important but rather underutilized resource with great potential for consumption as food. In Soviet territories, wild plant use knowledge was particularly relevant during times of food shortages caused by economic, political, and social factors. Therefore, the production of new books was probably an attempt of ostentation of wild food diversity of Soviet Union and, above all, the result of increasingly clear centralization as one of the dimensions of general Russification.

The impact of war and politics on book knowledge circulation of wild food plants and their uses is still of particular research interest today. Many wild plants in the studied data sample were recommended as substitutes for other products or ingredients, correlated with food supply problems in the USSR. On average, wartime books had fewer wild plant species recommended for consumption and less variety in the food uses proposed compared to post-war books, which is related to not only the need for staple foods (such as main dishes) during the war, but also the specificity of the literature and the lack of resources for printing.

The popularization of wild food plants in the Soviet Union during World War II and later when the economy started to collapse may have had an influence on the circulation of knowledge regarding the use of wild food plants, especially in the last decade of the Soviet Union, contributing to the centralization of knowledge production. This combination of findings provides some support for the conceptual premise that future studies on the current topic are needed. Additional study with a greater focus on the actors involved in book knowledge circulation is suggested. In order to establish if there is a correlation between actual use and a homogenization effect, a comparison with field studies conducted in regions potentially covered by the distribution of books is advisable.

## Figures and Tables

**Table 1:** Books on wild food plant use published in the Soviet Union. Highlighted in green are the books used for this study.

Author(s)	Year	Title (Translated Title)	Place of Publication	Print Run
Keller [39]	1941	*“Дикие съедoбные растения” (“*Wild edible plants*”)*	Moscow, Leningrad	50,000
Bosse [40]	1942	*“Гoтoвьте из диких весенних растений. Мучные изделия, супы, салаты” (“*Cook from wild spring plants. Pastries, soups, salads*”)*	Moscow	50,000
Dmitriyevsky and Semenov, [41]	1942	*“Прoстейшие спoсoбы сушки oвoщей, бoтвы и грибoв” (“*The simplest ways to dry vegetables, shoots and mushrooms*”)*	Leningrad	5000
Gollerbakh et al. [42]	1942	*“Главнейшие дикoрастущие пищевые растения Ленинградскoй oбласти” (“*The main wild food plants of the Leningrad Region*”)*	Leningrad	-
Korchagin [43]	1942	*“Чай и кoфе из культурных и дикoрастущих растений Ленoбласти” (“*Tea and coffee from cultivated and wild plants in the Leningrad region*”)*	Leningrad	3000
Krasilnikov [44]	1942	*“Витамин С в хвoе и листьях деревьев и кустарникoв” (“*Vitamin C in the needles and leaves of trees and shrubs*”)*	Leningrad	3000
Rozhevits [45]	1942	*“Испoльзуйте для питания прибрежную и вoдную растительнoсть” (“*Use coastal and aquatic vegetation for food*”)*	Leningrad	3000
Troitskaya et al. [46]	1942	*“Испoльзoвание в пищу дикoрастущих съедoбных растений” (“*The use of wild edible plants for food*”)*	Leningrad	-
Zorin and, Ivanenko [47]	1942	*“Примитивные спoсoбы выпечки хлеба в пoлевых услoвиях” (“*Primitive ways of baking bread in the field*”)*	Kalinin	2000
Pankova and Nikitin [48]	1943	*“Пригoтoвление пищи из бoтвы и дикoрастущих съедoбных растений” (“*Cooking with shoots and wild edible plants*”)*	Leningrad	5000
Sokolov [49]	1943	*“Как oбеспечить себя витаминoм C в зимнее время” (“*How to ensure you get enough vitamin C in winter*”)*	Leningrad	5000
Krasinski [50]	1944	*“Сахар из мoжжевелoвoй ягoды” (“*Juniper berry suga*r”)*	Moscow	15,000
Muller [51]	1944	*“О витаминах” (“*About the vitamins*”)*	Leningrad	3000
Nikitin and Pankova [52]	1944	*“Дикoрастущие съедoбные растения” (“*Wild edible plants*”)*	Leningrad	1500
Verzilin [31]	1946	*“Пo следам Рoбинзoна” (“*In Robinson’s footsteps*”)*	Moscow, Leningrad	45,000
Verzilin. [33]	1953	*“Пo следам Рoбинзoна” (“*In Robinson’s footsteps*”)*	Moscow, Leningrad	100,000
Verzilin [34]	1956	*“Пo следам Рoбинзoна” (“*In Robinson’s footsteps*”)*	Leningrad	75,000
Rybitskiy and Gavrilov [53]	1969	*“Дикoрастущие плoды и ягoды” (“*Wild fruits and berries*”)*	Leningrad	150,000
Koscheev [54]	1981	*“Дикoрастущие съедoбные растения в нашем питании” (“*Wild edible plants in our diet*”)*	Moscow	300,000
Verzilin [35]	1982	*“Пo следам Рoбинзoна” (“*In Robinson’s footsteps*”)*	Minsk	-
Zuyev [55]	1988	*“Дары русскoгo леса” (“*Gifts of the Russian forest*”)*	Moscow	315,000
Pitenev [56]	1989	*“Лесная кухня” (“*Woodland cuisine*”)*	Novosibirsk	100,000
Berson [57]	1991	*“Дикoрастущие съедoбные растения” (“*Wild edible plants*”)*	Leningrad	160,000
Koscheev. [58]	1991	*“Напитки из дикoрастущих плoдoв и ягoд” (“*Wild fruit and berry drinks*”)*	Moscow	200,000

**Table 2:** The 29 most popular taxa in the two research periods.

Taxa	War DUR	Taxa	Post-War DUR
*Aegopodium podagraria* L.	28	*Carum carvi* L.	22
*Anthriscus* sp.	19	*Crataegus* sp.	26
*Arctium* sp.	21	***Epilobium angustifolium* L.**	36
*Butomus umbellatus* L.	12	*Fragaria vesca* L.	45
*Capsella bursa-pastoris* (L.) Medik.	9	*Fragaria viridis* Weston	21
*Chenopodium album* L.	38	*Hippophae rhamnoides* L.	36
***Epilobium angustifolium* L.**	19	*Origanum vulgare* L.	25
*Geum urbanum* L.	8	*Prunus spinosa* L.	21
*Heracleum sibiricum* L.	28	*Pyrus communis* subsp. *communis*	20
*Hylotelephium maximum* (L.) Holub	10	*Ribes nigrum* L.	40
*Hylotelephium telephium* subsp. *telephium*	10	*Ribes rubrum* L.	29
*Lamium album* L.	8	*Rosa* sp.	54
*Leymus arenarius* (L.) Hochst.	18	*Rosa acicularis* Lindl.	26
*Nymphaea alba* L.	7	*Rosa davurica* Pall.	26
*Oxalis acetosella* L.	11	*Rosa laxa* Retz.	25
*Phragmites australis* subsp. *australis*	18	*Rosa pendulina* L.	25
*Plantago major* L.	10	*Rubus caesius* L.	34
***Rumex acetosa* L.**	29	*Rubus chamaemorus* L.	33
*Sagittaria sagittifolia* L.	11	*Rubus idaeus* L.	48
*Stellaria media* (L.) Vill.	12	*Rubus saxatilis* L.	40
*Symphytum* sp.	8	***Rumex acetosa* L.**	20
*Taraxacum sect. Taraxacum* F.H.Wigg.	17	*Sambucus nigra* L.	22
*Trifolium hybridum* L.	8	*Sorbus aucuparia* L.	56
*Trifolium pratense* L.	8	***Typha latifolia* L.**	21
*Trifolium repens* L.	8	*Vaccinium myrtillus* L.	46
***Typha latifolia* L.**	10	*Vaccinium oxycoccos* L.	59
*Urtica* sp.	8	*Vaccinium uliginosum* L.	28
*Urtica dioica* L.	15	*Vaccinium vitis-idaea* L.	57
*Urtica urens* L.	14	*Viburnum opulus* L.	28

**Table 3:** Number of records of specific food uses in the sources during WWII (1941–1943) and after WWII (1953–1989) in the USSR.

Period/No of DURs Related to	Desserts	Main Dishes	Substitutes	Alcoholic Drinks
During WWII (1941–1943)	2	154	22	4
After WWII (1953–1989)	181	125	56	134

## Data Availability

https://doi.org/10.5281/zenodo.6638909.
